# A novel *fiber-2*-edited live attenuated vaccine candidate against the highly pathogenic serotype 4 fowl adenovirus

**DOI:** 10.1186/s13567-021-00907-z

**Published:** 2021-02-27

**Authors:** Quan Xie, Shiya Cao, Wei Zhang, Weikang Wang, Luyuan Li, Qiuqi Kan, Hui Fu, Tuoyu Geng, Tuofan Li, Zhimin Wan, Wei Gao, Hongxia Shao, Aijian Qin, Jianqiang Ye

**Affiliations:** 1 Key Laboratory of Jiangsu Preventive Veterinary Medicine, Key Laboratory for Avian Preventive Medicine, Ministry of Education, College of Veterinary Medicine, Yangzhou University, Yangzhou, 225009 Jiangsu China; 2 Jiangsu Co-Innovation Center for Prevention and Control of Important Animal Infectious Diseases and Zoonoses, Yangzhou, 225009 Jiangsu China; 3 Joint International Research Laboratory of Agriculture and Agri-Product Safety, the Ministry of Education of China, Yangzhou University, Yangzhou, 225009 Jiangsu China; 4 Institute of Agricultural Science and Technology Development, Yangzhou University, Yangzhou, 225009 Jiangsu China; 5 Sinopharm Yangzhou VAC Biological Engineering Co.Ltd, Yangzhou, 225127 Jiangsu China; 6 College of Animal Science and Technology, Yangzhou University, Yangzhou, 225009 Jiangsu China

**Keywords:** FAdV-4, CRISPR/Cas9, Recombinant virus, Attenuation, Vaccine candidate

## Abstract

Recently, the outbreaks of hydropericardium-hepatitis syndrome (HHS) caused by the highly pathogenic fowl adenovirus serotype 4 (FAdV-4) have resulted in huge economic losses to the poultry industry globally. Although several inactivated or subunit vaccines have been developed against FAdV-4, live-attenuated vaccines for FAdV-4 are rarely reported. In this study, a recombinant virus FA4-EGFP expressing EGFP-Fiber-2 fusion protein was generated by the CRISPR/Cas9 technique. Although FA4-EGFP shows slightly lower replication ability than the wild type (WT) FAdV-4, FA4-EGFP was significantly attenuated in vivo compared with the WT FAdV-4. Chickens infected with FA4-EGFP did not show any clinical signs, and all survived to 14 day post-infection (dpi), whereas those infected with FAdV-4 showed severe clinical signs with HHS and all died at 4 dpi. Besides, the inoculation of FA4-EGFP in chickens provided efficient protection against lethal challenge with FAdV-4. Compared with an inactivated vaccine, FA4-EGFP induced neutralizing antibodies with higher titers earlier. All these data not only provide a live-attenuated vaccine candidate against the highly pathogenic FAdV-4 but also give a potential insertion site for developing FAdV-4-based vaccine vectors for delivering foreign antigens.

## Introduction

Fowl adenoviruses (FAdV) are non-enveloped viruses with a double-stranded DNA genome, belonging to *Adenoviridea* and genus *Aviadenovirus* [1]. Based on the profile of restriction enzyme digestion and the sera cross-neutralization assay, FAdV has been clustered into 5 species (FAdV-A ~ E) with 12 serotypes (FAdV-1 to 8a and 8b to 11) [2]. The infection of FAdV mainly causes clinical symptoms, including inclusion body hepatitis (IBH), hepatitis-hydropericardium syndrome (HHS), and gizzard erosion and ulceration (GEU) [3]. Among the 12 serotypes of FAdV, FAdV-4 is the main causative agent for HHS in chickens [4–6]. It is noteworthy that the recent outbreaks of HHS caused by the highly pathogenic FAdV-4 have resulted in huge economic losses to the poultry industry worldwide [7–10]. Many inactivated or subunit vaccines have been developed to control HHS [3]. However, the live-attenuated vaccine against FAdV-4 has rarely been reported [11, 12].

Unlike other serotypes, serotypes FAdV-1, FAdV-4, and FAdV-10 have two *fiber* genes (*fiber-1* and *fiber-2*) [13]. Recently, our group and Wang’s group found that Fiber-1, but not Fiber-2, directly triggered the viral infection of FAdV-4 via its shaft and knob domains [14, 15]. However, Zhang et al. reported that Fiber-2, but not Fiber-1, was identified as one of the major virulent determiners for the highly pathogenic FAdV-4 endemic in China [16]. These findings indicate that Fiber-1, but not Fiber-2, is required for the infection of FAdV-4, whereas Fiber-2, but not Fiber-1, plays vital roles in the pathogenicity of FAdV-4. Therefore, Fiber-2 might be an efficient target for developing live-attenuated vaccine candidates against FAdV-4. Here, we used the CRISPR/Cas9 approach to target the *fiber-2* gene of the wild type (WT) FAdV-4 to generate a recombinant virus FA4-EGFP expressing EGFP-Fiber-2 fusion protein. In vitro and in vivo studies demonstrated that FA4-EGFP is highly attenuated compared with the WT FAdV-4 and could provide efficient protection against lethal challenge with WT FAdV-4.

## Materials and methods

### Cells, viruses, and antibodies

The FAdV-4 strain SD was isolated and stored in our laboratory and propagated in leghorn male hepatoma (LMH) cells. LMH cells were purchased from ATCC and cultured in Dulbecco Modified Eagle Medium/F12 (Gibco, NY, USA) supplemented with 10% fetal bovine serum (Lonsera, Shanghai, China) in a 5% CO_2_ incubator at 37 ℃. Monoclonal antibody (mAb) 3B5 against Fiber-1, and mAb 3C2 against Fiber-2 were generated in our laboratory, and mAb 3C2 against Fiber-2 were generated in our laboratory, and Professor Hongjun Chen kindly provided mAb 1C9 against Fiber-2.

### Construction of sgRNA and donor plasmids

The sgRNA targeting *fiber-2* of the FAdV-4 genome was designed using the CRISPR guide RNA designing website [17] and cloned into the CRISPR/Cas9 vector lentiCRISPR v2. The sequences of the sgRNA are listed in Table 1. The donor plasmid containing the EGFP sequence at the N terminus of *fiber-2* was constructed by several rounds of overlapping PCR. The homologous arm (HA) designed at both ends was 1000 bp in length, respectively. The template was assembled as the HAL-EGFP-F2-HAR and finally cloned into the pMD19 simple vector. The primers used for constructing donor plasmid are provided in Table 2.

**Table 1:** List of primers used for sgRNA cloning

	Sequences of primers (5′–3′)
sgRNA1	F: CACCGGGTTTATCCTTTCGATTACG
	R: AAACCGTAATCGAAAGGATAAACCC
sgRNA2	F: CACCGCGTGCTCTACAGCTGTCCAG
	R: AAACCTGGACAGCTGTAGAGCACGC

**Table 2:** PCR primers for constructing donor plasmid and detecting the recombinant virus

PCR products	Sequences of primers (5′–3′)
HAL	F: GGTGACCTACTGACCCTCAACACC
	R: CAGCTCCTCGCCCTTGCTCACCATTGTTCCCGTTGGGGGA
EGFP	F: TCCCCCAACGGGAACAATGGTGAGCAAGGGCGAGGAGCTG
	R: CTTCTTTTAGGGGCCCGGAGCTTGTACAGCTCGTCCATG
*fiber-2* + HAR	F: CATGGACGAGCTGTACAAGCTCCGGGCCCCTAAAAGAAG
	R: CTACTTTACCTGCATTTCGTCAG
Partial *fiber-2*	F: CTCCAACTGGTTTGACCAGAACG
	R: GTCAAGCTGGGATGCTCTCACCATGC

### Generation of the recombinant FAdV-4-EGFP

LMH cells were transfected with the sgRNA targeting both ends of the *fiber-2* gene with 2 μg of each sgRNA 24 h post-transfection (hpt), the LMH cells were infected with FAdV-4 at 0.1 MOI and then were transfected with 4 μg of the donor plasmid. The infected LMH cells were observed through a fluorescence microscope 24 hours post-infection (hpi). The recombinant virus, designated as FA4-EGFP, was purified by limiting the dilution assay and virus plaque assay. The purified virus was further identified by Western blot, PCR, and sequencing.

### Growth curve of the FAdV-4-EGFP in LMH cells

To determine the replication capacity of the recombinant virus FA4-EGFP, LMH cells seeded in a 6-well plate (about 1.2 × 10^6^ cells per well) were infected with WT FAdV-4 and the recombinant virus FA4-EGFP at 0.1 MOI, respectively, and then the viruses were harvested at 24, 48, 72, 96 and 120 hpi, and stored at −80 ℃ until use. The TCID_50_ of the harvested viruses were determined in 96-well plates by serial dilution from 10^–1^ to 10^–8^, and detected at 72 hpi by IFA and calculated by the Reed-Muench method.

### Western blot assay

The LMH cells infected with FAdV were collected and lysed in lysis buffer (CST, MA, USA) with PMSF (Beyotime, Shanghai, China), protease and phosphatase inhibitors (CST, MA, USA). The lysates were boiled in the loading buffer and were then subjected to 10% SDS-PAGE and transferred to nitrocellulose (NC) membranes (GE Healthcare Life sciences, Freiburg, Germany). After blocking with 5% skimmed milk in PBST for 1 h at room temperature (RT), the membranes were reacted with the corresponding antibodies at 4 ℃ overnight. After being washed with PBST three times, the membrane was incubated with HRP-labelled secondary antibodies for 1 h at RT. After another three washes, the membranes were developed with chemiluminescent reagents and imaged with an automatic imaging system (Tanon 5200).

### Indirect immunofluorescent assay

The LMH cells infected with viruses were fixed with pre-chilled acetone: ethanol (3:2 v/v) mixture for 5 min at RT and washed with PBS. The cells were then incubated with the diluted mAb 3B5 against Fiber-1 for 45 min at 37 ℃. After washing three times with PBS, the cells were incubated with the diluted second antibody (goat anti-mouse IgG-FITC) for another 45 min at 37 ℃. Again, after three washes with PBS, the cells were observed by invert fluorescence microscopy.

### Animal experiments

For the pathogenesis analysis, a total of 120 one-day-old SPF chickens were randomly divided into three groups (40 chickens per group; Positive control group: chickens infected with FAdV-4; Experiment group: chickens infected FA4-EGFP; Negative control group: chickens inoculated with 1% culture medium). The chickens were housed in different negative-pressure isolators for 14 days and subsequently infected with 10^6^ TCID_50_ of the indicated virus in 200 μL of 1% culture medium intramuscularly, the negative control group was inoculated with the same volume of 1% culture medium. On 2, 3, 4, 5, 6 dpi, the cloacal swabs were collected, and three chickens in each group were euthanized; the liver, the spleen, the kidney was collected for viral titration or histopathology analysis. For the protective analysis, the chickens survived in the experimental group, and the negative control group at 21 dpi were challenged with 10^6^ TCID_50_ of FAdV-4 in 200 μL of 1% culture medium intramuscularly. On 2, 3, 4, 6, 8 days post-challenge (dpc), the cloacal swabs were collected, and three chickens in each group were euthanized; the liver, the spleen, the kidney was collected for viral titration. The clinical symptoms and mortality of the infected or challenged chickens were monitored daily.

To further compare the protective efficacy of FA4-EGFP with the inactivated FAdV-4 vaccine candidate, a total of 50 2-week old SPF chickens were randomly divided into 5 groups. Three groups were inoculated intramuscularly with the three doses of FA4-EGFP (10^6^ TCID_50_, 10^5^ TCID_50_, 10^4^ TCID_50_) in 200 μL of 1% culture medium, respectively. The other two groups were inoculated with the inactivated oil-emulsion FAdV-4 containing 5 × 10^6^ TCID_50_ of viruses and the same emulsion volume intramuscularly, respectively. On 7, 14, 21 dpi, the blood was collected for the neutralization test. On 21 dpi, chickens were challenged with 10^6^ TCID_50_ of FAdV-4 in 200 μL of 1% culture medium, and the survival data was recorded. The clinical symptoms and mortality of the challenged chickens were monitored daily.

All animal experiments were performed following the “Guidelines for Experimental Animals” and the protocol (SYXY-19), which was approved by the Animal Care and Use Committee of Yangzhou University (Yangzhou, China). At the end of the experiment, all the chickens were euthanized by CO_2_.

### Neutralization test

Different chicken sera dilutions were first mixed with 1000 TCID_50_ of FAdV-4 and incubated for 1 h at 37 ℃. The mixtures were then added to the 96-well plate with LMH cells and incubated for 2 h at 37 ℃. After washing once, the cells were cultured in F12/DMEM medium with 1% fetal bovine serum. After being cultured for 96 h, the cells were fixed and subjected to IFA analysis using mAb 3B5 against Fiber-1 of FAdV-4 as previously described.

### Titration of viral titer in organs and cloacal swabs

The liver, spleen, and kidney collected were homogenized, treated with tenfold penicillin and streptomycin for 1 h, and centrifuged to obtain the supernatant. The cloacal swabs collected from the chickens were placed in 800 μL of PBS. After three times of freeze–thaw cycles, the samples were treated the same with organ homogenates. The virus-containing supernatants were then serially diluted and inoculated into LMH cells. The infected LMH cells were fixed and detected by IFA using 3B5 mAb against Fiber-1 4 dpi, and the TCID_50_ of these supernatants was determined by the Reed-Muench method.

### Statistical analysis

All the results are presented as means ± standard deviation. This study’s statistical analysis was performed with a Student test or One-way ANOVA test using GraphPad 5 software. *P*-value of < 0.05 was considered significant. *, **, and *** indicate *P*-value less than 0.05, 0.01 and 0.001, respectively.

## Results

### Generation of recombinant virus FA4-EGFP expressing EGFP-Fiber-2 protein

**Figure 1:**
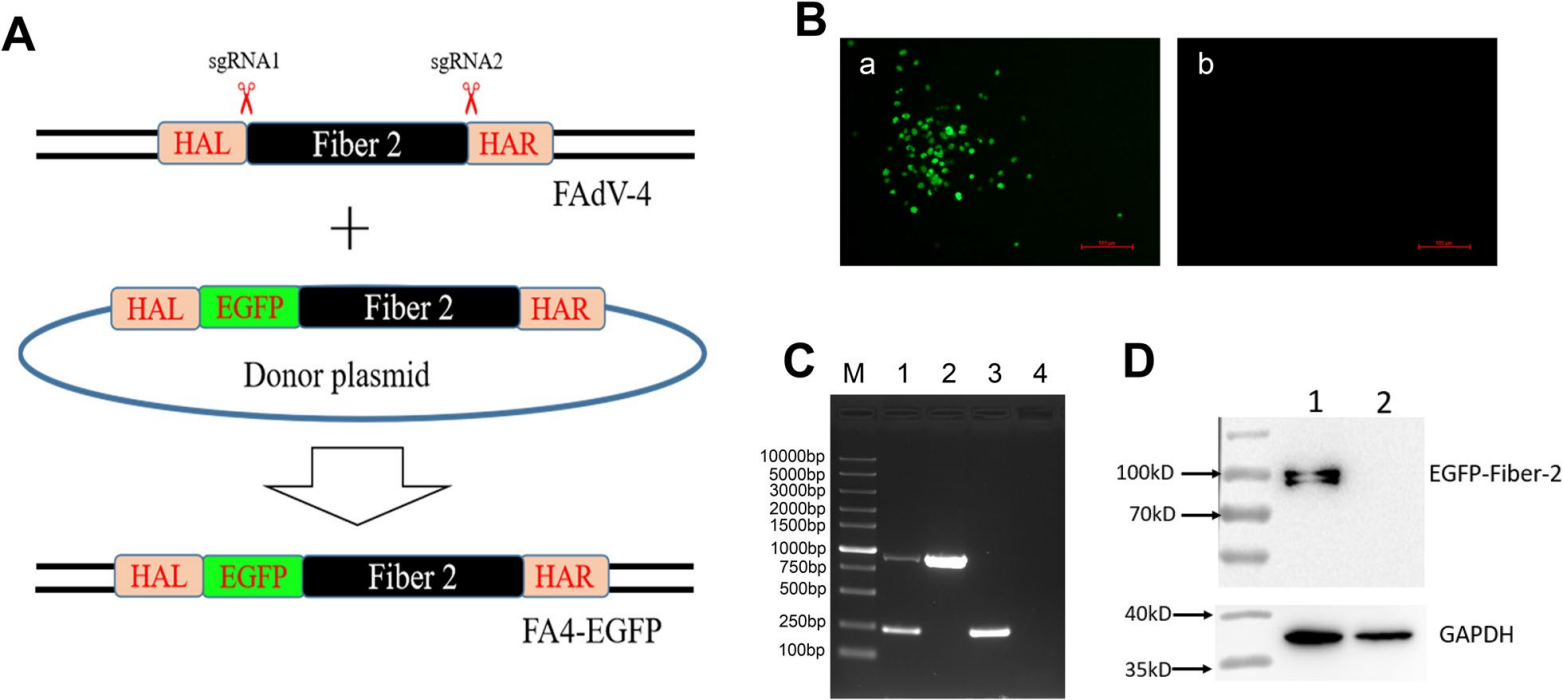
**Generation and identification of the recombinant virus FA4-EGFP.** **A** The homology-dependent knock-in strategy for generating the recombinant virus FA4-EGFP using the CRISPR/Cas 9 system. LMH cells were first transfected with sgRNA1 and sgRNA2. 24 hpt, the LMH cells were infected with FAdV-4 and transfected with donor plasmid. The recombinant virus FA4-EGFP was then purified by limiting dilution assay and viral plaque assay. **B** 24 hpt, the EGFP could be found by fluorescence microscopy in the transfected LMH cells with the infection of FAdV-4 at 24 hpi (**a**), whereas no EGFP could be observed in the FAdV-4 infected LMH cells without the transfection of sgRNA and donor plasmid (**b**). **C** PCR identification of the recombinant virus FA4-EGFP. The unpurified recombinant virus FA4-EGFP (Lane 1), the purified FA4-EGFP (Lane 2), and the WT FAdV-4 (Lane 3) were detected using specific primers listed in Table 2. **D** Western blot analysis of the recombinant virus FA4-EGFP. LMH cells infected with the purified recombinant virus FA4-EGFP (Lane 1) and the WT FAdV-4 (Lane 2) were harvested and lysed, and the lysates were then tested with Western blot by monoclonal antibodies against GFP.

To evaluate whether the *fiber-2* gene of FAdV-4 could be an efficient target for developing live-attenuated vaccine candidates against FAdV-4 or vaccine vectors for expressing foreign antigens, two sgRNA targeting N- and C-terminus of *fiber-2*, respectively, were first designed and cloned into lentiCRISPR v2, and the donor plasmid containing the EGFP sequence at the N terminus of *fiber-2* was constructed by overlap PCR as described in Figure 1A. A recombinant virus, designated as FA4-EGFP expressing EGFP-Fiber-2 fusion protein, was generated through the transfection of the two sgRNA and the donor plasmid and the infection of FAdV-4 in LMH cells. Notably, the virus plagues with EGFP could be found in the transfected LMH cells with the infection of FAdV-4 at 24 hpi, whereas no EGFP could be observed in the FAdV-4 infected LMH cells without the transfection of sgRNA and donor plasmid (Figure 1B), indicating the recombinant virus FA4-EGFP was efficiently generated. After serial limit dilution and plague purification, the purified FA4-EGFP was further identified by sequencing, Western blot, and PCR. As shown in Figure 1C, the partial *egfp-fiber-2* fusion gene was amplified in the purified FA4-EGFP, but not in FAdV-4. Both partial *egfp-fiber-2* fusion gene and partial *fiber-2* gene were amplified in the unpurified FA4-EGFP, whereas only partial *fiber-2* gene was amplified in FAdV-4 using specific primers as listed in Table 2. In the Western blot, the EGFP-Fiber-2 fusion protein was efficiently detected in the LMH cells infected with the purified FA4-EGFP as described in Figure 1D. The conformed sequence of the recombinant GFP-Fiber-2 in FA4-EGFP are provided as Additional file 1. All these data demonstrate that a novel recombinant virus FA4-EGFP expressing EGFP-Fiber-2 protein is generated.

### FA4-EGFP replicated slower than the WT FAdV-4 in vitro

**Figure 2:**
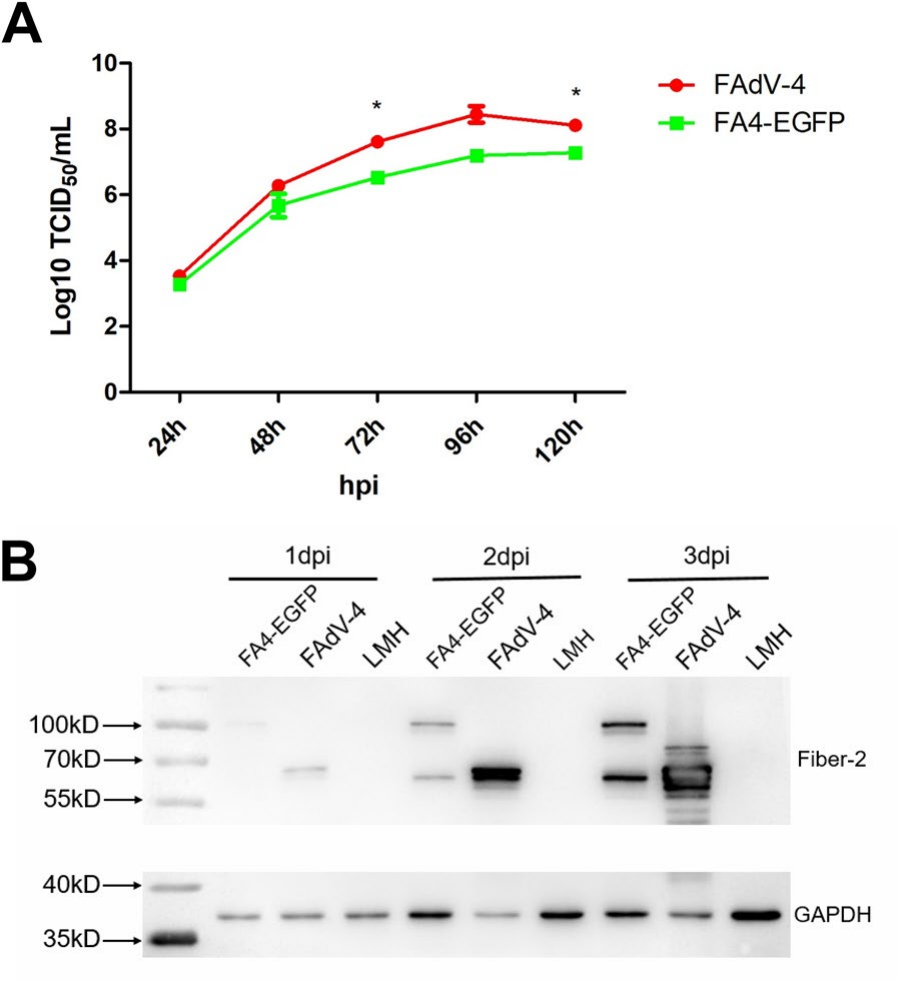
**The recombinant virus FA4-EGFP replicated slower than FAdV-4 in vitro.** **A** LMH cells were infected with FA4-EGFP and FAdV-4 at the same dose, respectively, the viral supernatant collected from the infected LMH cells at the indicated time points were then titrated by TCID_50_. This experiment was done in triplicate and repeated twice. **B** LMH cells infected with FA4-EGFP and FAdV-4 were harvested at different time points, the lysates of these infected LMH cells were then tested by Western blot with monoclonal antibody 1C9 specific to Fiber-2.

To compare the growth kinetics of the recombinant virus FA4-EGFP with the WT FAdV-4, LMH cells were infected with the same dose of FA4-EGFP and FAdV-4, respectively, and then the virus supernatants from the infected LMH cells at different time points were collected and titrated. As described in Figure 2A, although FA4-EGFP could efficiently grow in LMH cells, FA4-EGFP replicated slightly slower than WT FAdV-4. Notably, the peak titer of FAdV-4 could reach 10^8^ TCID_50_/mL while the FA4-EGFP could only reach 10^7^ TCID_50_/mL within 5 days. This TCID_50_ data for the growth kinetics was also confirmed by Western blot analysis. As shown in Figure 2B, the Fiber-2 protein with a strong strip in the LMH cells infected with FAdV-4 could be efficiently detected at 48 hpi and 72 hpi, whereas the band of the Fiber-2 protein in the LMH cells infected with FA4-EGFP was weak. All these demonstrate that although the replication ability of FA4-EGFP is weaker than that of WT FAdV-4, FA4-EGFP can efficiently replicate in LMH cells with high viral titer.

### FA4-EGFP was highly attenuated in vivo

**Figure 3:**
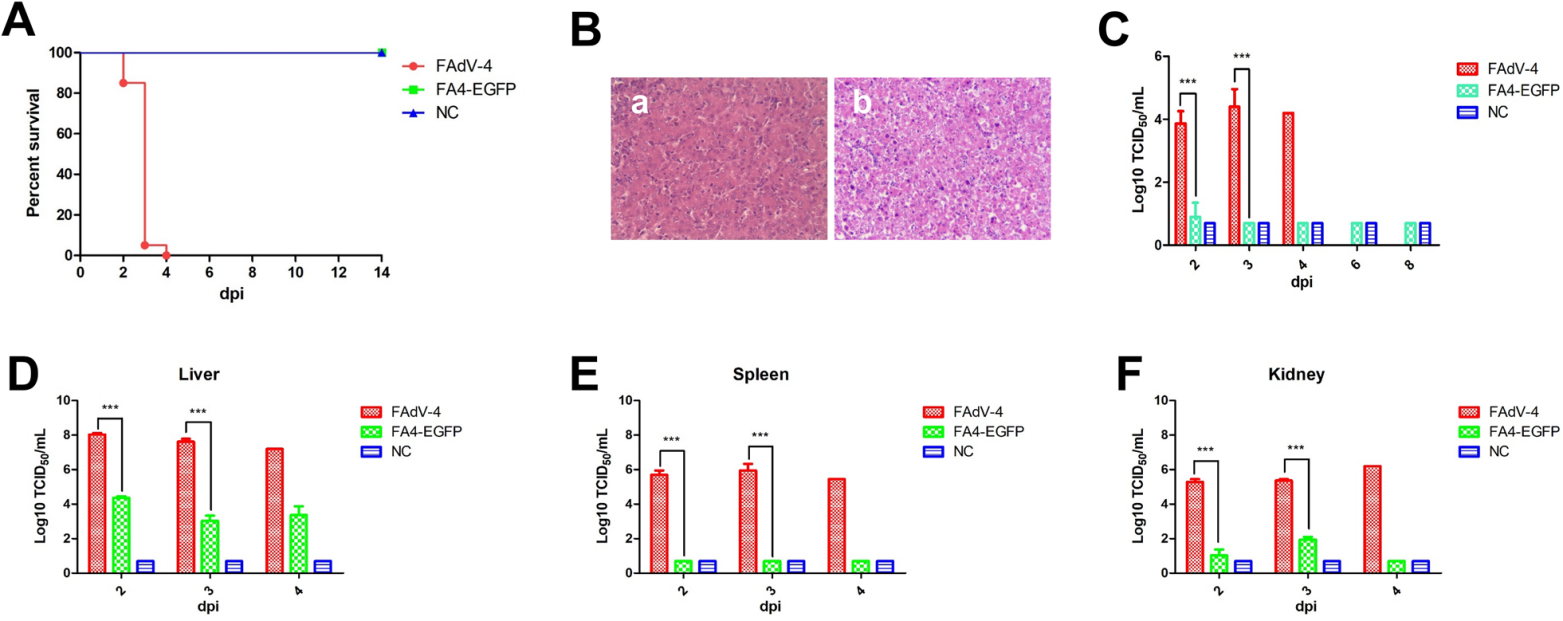
**Pathogenicity of the recombinant virus FA4-EGFP in vivo.** SPF chickens were randomly divided into three groups and then infected with FA4-EGFP, WT FAdV-4, and 1% culture medium, respectively. The clinical symptoms and mortality of the infected chickens were monitored daily, and the liver, spleen, kidney, and cloacal swabs were collected for viral titration at the indicated time points. **A** Percent of survival for these infected chickens. **B** Representative histological changes in liver tissues from chickens infected with FA4-EGFP (**a**) and wild-type FAdV-4 (**b**). Viral shedding in cloacal swabs from chickens of different groups. **C** Viral loads in the liver (**D**), spleen (**E**), and kidney (**F**) tissues from these infected chickens of different groups.

To evaluate the viral replication and pathogenesis of FA4-EGFP in vivo, SPF chickens were infected with FA4-EGFP and wild-type FAdV-4 at the same dose. The infected chickens' clinical symptoms and mortality were monitored daily, and the liver, spleen, kidney, and cloacal swabs were collected for viral titration at the indicated time points. At 2 dpi, the chickens infected with FAdV-4 began to show clinical signs characterized by depression, sleepiness and huddling together with ruffled feathers, and all the chickens died within 4 dpi as described in Figure 3A. However, all the chickens infected with FA4-EGFP did not show any clinical signs and death throughout the experiment (Figure 3A). Necropsy analysis demonstrates that the chickens infected with FAdV-4 show HHS, whereas no gross lesion in the heart, liver, spleen, or kidney was observed in the chickens infected with FA4-EGFP (data not shown). The histopathological analysis further demonstrated that the degeneration and necrosis of hepatocytes and the intranuclear inclusion bodies in hepatocytes were observed in the chickens infected with FAdV-4, whereas no obvious histopathological symptoms were found in the chickens infected with FA4-EGFP as shown in Figure 3B. For virus shedding, as shown in Figure 3C, the high viral titer about 10^4^–10^5^ TCID_50_/mL was detected at 2–4 dpi in the cloacal swab samples from chickens infected with FAdV-4, whereas the low viral titer was detected at 2 dpi in the cloacal swab samples of chickens infected with FA4-EGFP. Chickens in the FA4-EGFP group did not shed any viruses in cloaca at 3–8 dpi. For virus loading in tissues, as described in Figure 3D, the viral titers in the liver from the chickens infected with FAdV-4 reached 10^7^–10^8^ TCID_50_/mL at 2–4 dpi, whereas those in chickens infected with FA4-EGFP were only about 10^2^–10^4^ TCID_50_/mL.

Similarly, as shown in Figures 3E, F, a high viral titer of FAdV-4 was detected in the spleen and kidney, whereas a very low titer of FA4-EGFP was detected in the kidney, and no virus was detected in the spleen. These data from the virus cloacal shedding and tissue loading were consistent with the clinical signs and survival data. All these reveal that the FA4-EGFP virus is highly attenuated in chickens.

### FA4-EGFP provides efficient protection against lethal challenge of FAdV-4

**Figure 4:**
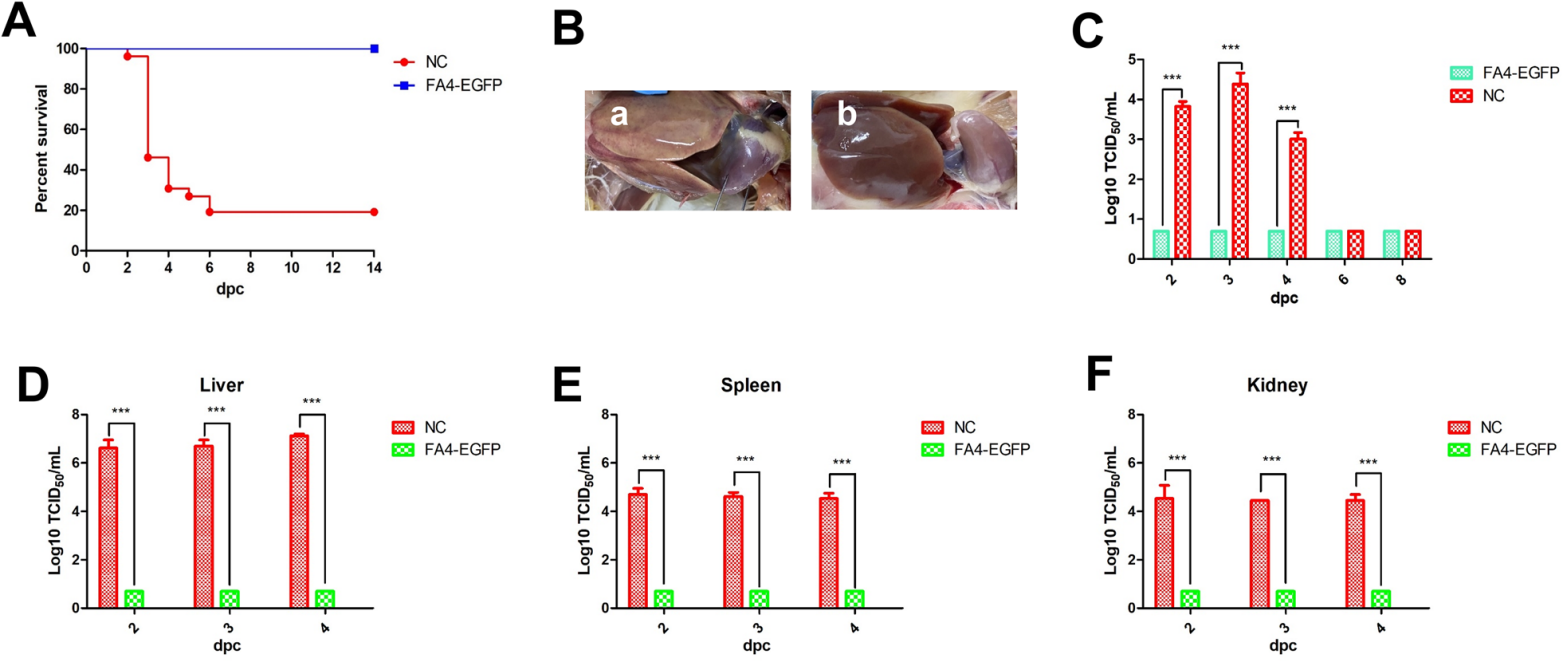
**FA4-EGFP provided efficient protection against lethal challenge.** The SPF chickens inoculated with FA4-EGFP were challenged at 21 dpi with the lethal dose of FAdV-4. The clinical symptoms and mortality of the challenged chickens were monitored daily, and the liver, spleen, kidney, and cloacal swabs were collected for viral titration at the indicated time points. **A** Percent of survival for the challenged chickens. **B** The representative gross lesion in the heart and liver from the challenged control chickens (**a**) and the challenged chickens previously inoculated with FA4-EGFP (**b**). Viral shedding in cloacal swabs from the challenged chickens. **C** Viral loads in the liver (**D**), spleen (**E**), and kidney (**F**) tissues from the challenged chickens.

To evaluate the protective efficacy of the recombinant virus FA4-EGFP, the chickens previously inoculated with FA4-EGFP were challenged with a lethal dose of FAdV-4 at 21 dpi. The clinical signs and mortality of the challenged chickens were monitored daily, and the liver, spleen, kidney, and cloacal swabs were collected for viral titration at different time points post-challenge. After the challenge, the control group’s chickens exhibited clinical signs, including depression, loss of appetite, and huddling together with ruffled feathers. These challenged control chickens’ mortality reached 80% (21/26) at day 6 post-challenge (Figure 4A), whereas the challenged chickens previously inoculated with FA4-EGFP did not show any clinical symptoms, and all survived. Besides, necropsy analysis shows that hydropericardium and hepatitis were found in the challenged control group, but not in the challenged chickens previously inoculated with FA4-EGFP (Figure 4B). Moreover, high viral titers in cloacal swabs, the liver, spleen, and kidney from the challenged control group were detected at 2–4 dpc (post-challenge), whereas no virus could be detected at all the time points tested in the challenged chickens previously inoculated with FA4-EGFP in cloacal swabs, liver, spleen, and kidney. All these results demonstrate that FA4-EGFP can be an efficient vaccine candidate against the highly pathogenic FAdV-4.

### FA4-EGFP induced more robust neutralizing activity than inactivated vaccine

**Figure 5:**
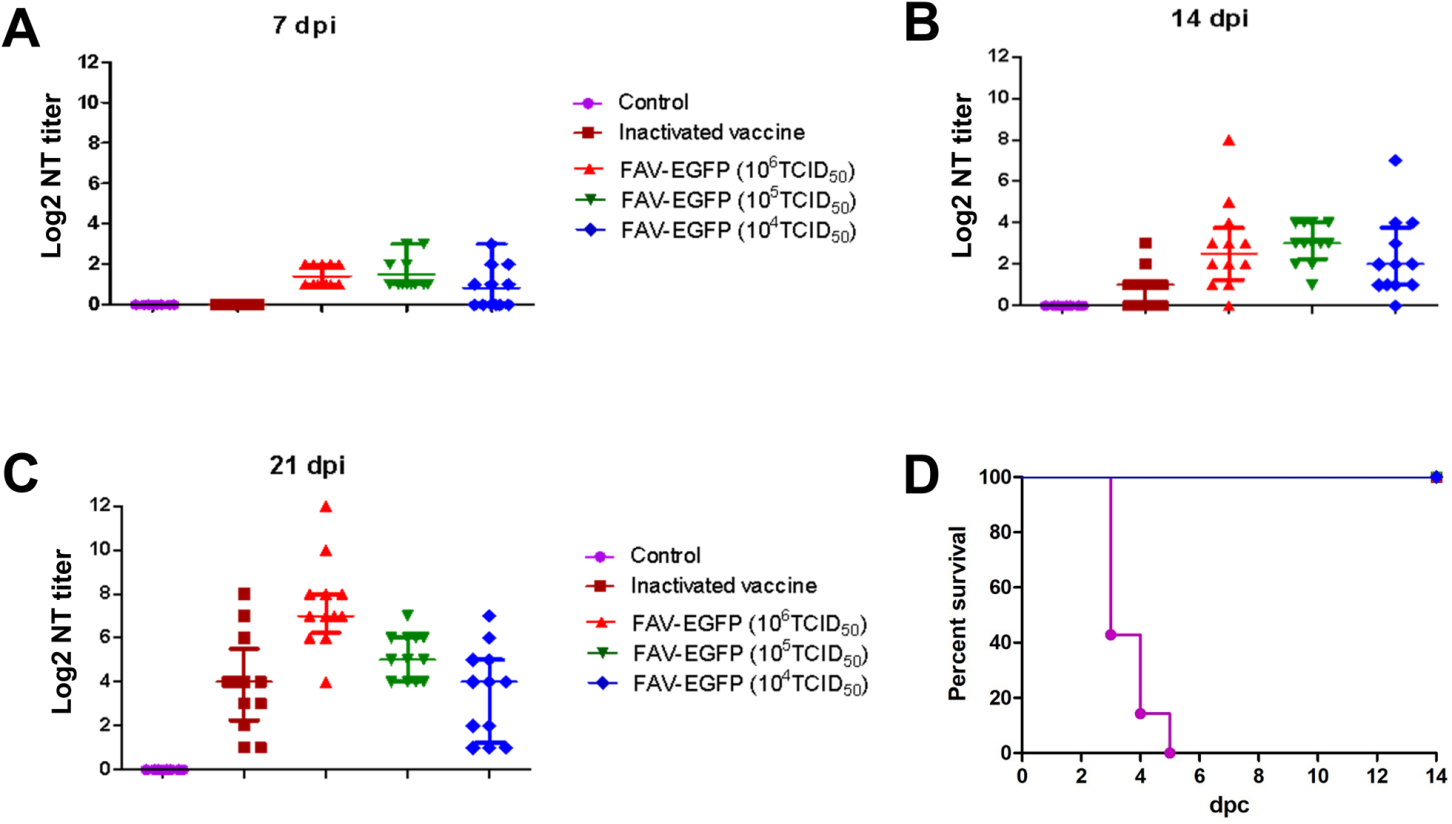
**FA4-EGFP induced higher robust neutralizing activity than the inactivated vaccine.** SPF chickens randomly divided into 5 groups were immunized with inactivated vaccine or infected with different doses of the recombinant virus FA4-EGFP. The neutralizing activity (NT) of sera from the inoculated chickens were tested at 7 dpi (**A**), 14 dpi (**B**) and 21 dpi (**C**), and then these chickens were challenged with the lethal dose of FAdV-4 at 21 dpi. **D** Percent of survival for the challenged chickens.

To further compare the protective efficacy of FA4-EGFP with the inactivated FAdV-4 vaccine candidate, 2-week old SPF chickens were inoculated with either one of the three doses of FA4-EGFP (10^6^ TCID_50_, 10^5^ TCID_50,_ 10^4^ TCID_50_), or the inactivated FAdV-4 containing 5 × 10^6^ TCID_50_ of viruses, respectively. The neutralizing activity (NT) of sera from the inoculated chickens were tested, and then these chickens were challenged with the lethal dose of FAdV-4 at 21 dpi. As described in Figure 5A, the average NT titer for sera at 7 dpi was 1.5, 1.8, and 1.3 in chickens inoculated with 10^6^ TCID_50_, 10^5^ TCID_50,_ and 10^4^ TCID_50_ of FA4-EGFP respectively, whereas that in the control chickens and chickens inoculated with the inactivated vaccine could not be detected. The average NT titer for sera in chickens inoculated with 10^6^ TCID_50_, 10^5^ TCID_50,_ and 10^4^ TCID_50_ of FA4-EGFP was 2.8, 3.0, and 2.3 at 14 dpi, respectively, and 7.5, 5.2, and 3.5 at 21 dpi, respectively, whereas that in the chickens inoculated with the inactivated vaccine was 0.8 at 14 dpi and 3.9 at 21 dpi as shown in Figures 5B, C. Moreover, inoculation with FA4-EGFP or the inactivated FAdV-4 provided efficient protection against lethal challenge at 21 dpi, whereas the challenged control chickens died at 5 dpc as described in Figure 5D. These data demonstrate that except for efficient protection, FA4-EGFP induces neutralizing antibodies with higher titers earlier than the inactivated FAdV-4 vaccine candidate.

## Discussion

The development of vaccines is one of the most efficient strategies to prevent and control diseases. Since the first outbreak of FAdV-4 in 1987 [18], various vaccine candidates, including inactivated [19–22], live-attenuated [11, 23–25], and recombinant subunit vaccines [26–30], have been generated. Different vaccines have unique advantages and disadvantages. Although the inactivated vaccine is highly safe and protective, the preparation of a high titer of the inactivated FAdV-4 is highly expensive. The cost for the generation of subunit vaccine is less expensive; however, the subunit vaccine’s protective efficacy is generally limited compared with the whole inactivated FAdV-4 vaccines. Notably, both the inactivated vaccine and the sub-unit vaccine mainly induce a humoral immune response, but not a T cell immune response. Live-attenuated vaccines can trigger humoral and cellular immune responses to provide consistent and strong immune protection against viruses. Mansoor et al. [23] and Schonewille et al. [11] used chicken embryos and QT-35 cells to passage the pathogenic FAdV-4 to generate live-attenuated FAdV-4 vaccine candidates. Recently, Grgic et al. [24] used a non-pathogenic FAdV-4 isolate as a live-attenuated FAdV-4 vaccine candidate. Although these live-attenuated FAdV-4 vaccine candidates could provide efficient protection against the pathogenic FAdV-4, the molecular mechanism for the attenuation of these adapted strains or non-pathogenic strains need to be further elucidated. In this study, we targeted the *fiber-2* gene, one of the virulent determiners for the pathogenesis of FAdV-4, to generate a recombinant virus FA4-EGFP carrying the EGFP-Fiber-2 fusion protein. Our in vitro and in vivo studies demonstrate that FA4-EGFP not only was highly attenuated but also could provide full protection against lethal challenge with the highly pathogenic FAdV-4.

The CRISPR/Cas 9 technique has been widely used to edit the host genes and modify the virus genes [31–34]. This study combined the homology-dependent knock-in and the CRISPR/Cas 9 approach to generate the recombinant virus FA4-EGFP. During the rescuing of the recombinant virus FA4-EGFP, the virus plagues with EGFP could be observed at 24 hpi in the transfected LMH cells with the infection of FAdV-4, highlighting that the strategy used in this study to generate recombinant FAdV-4 was very efficient, and the N-terminus of the *fiber-2* gene could be an efficient insertion site for expressing the foreign gene. Moreover, we found that the purified FA4-EGFP passaged in LMH cells for more than 10 passages was very stable with EGFP-Fiber-2 fusion protein expression and without the reversion into the WT FAdV-4 (Data not shown). Also, we used PCR to detect the purity of FA-EGFP in the livers, kidneys and cloacal swabs of chickens infected the FA-EGFP. Only the specific band for FA-EGFP could be efficiently amplified whereas the band specific to the WT FAdV-4 could not be amplified in these samples (data not shown). All these demonstrate that the purity of FA-EGFP is robust. Notably, except for the large band of the EGFP-Fiber-2 protein, a small band was also detected in the LMH cells infected with the purified FAdV-4 by mAb against Fiber-2 in Western blot (Figure 2B). The molecular weight of the small band detected in LMH cells infected with FA4-EGFP was very similar to the small band of Fiber-2 (two bands with similar molecular weights) in LMH cells infected with WT FAdV-4. A sequence assay further revealed that a potential truncated ORF initiated at the nucleotide 94 of *fiber-2* was found, which has never been reported. The potential truncated ORF in *fiber-2* might encode the small band of detected Fiber-2. Of course, whether *fiber-2* can encode two proteins needs to be further elucidated.

Moreover, it should be noted that infection with a high dose (10^6^ TCID_50_) of FA4-EGFP did not cause any clinical signs and all the infected chickens survived, whereas all the chickens infected with the same dose of WT FAdV-4 demonstrated severe clinical signs with HHS and died at 4 dpi, which highlights the significant attenuation of FA4-EGFP. In comparison with the inactivated FAdV-4 vaccine, even 50 or 500 times fewer doses of the live FA4-EGFP can induce earlier and stronger neutralizing antibody with efficient protection against the lethal challenge with FAdV-4, highlighting the potential application of the live-attenuated FA4-EGFP for controlling the disease caused by FAdV-4 could significantly save the cost for the generation of the high dose of the inactivated FAdV-4. Although the induced neutralizing antibody by the live attenuated FA4-EGFP in this study may contribute to an efficient protection, the molecular mechanism for the protection against FAdV-4 needs to be further investigated. Notably, Schonewille et al. reported that the QT-35 adapted live FAdV-4 vaccine candidate could provide efficient protection without inducing detectable neutralizing antibodies [11]. Schachner et al. also found the recombinant Fiber-2 subunit vaccine could not produce neutralizing antibodies but provide 96% protection against the lethal challenge of FAdV-4 [27]. Therefore, whether an efficient protection against FAdV-4 requires the induced neutralizing antibody needs to be further elucidated.

In summary, this is the first demonstration of the generation of the highly attenuated recombinant virus FA4-EGFP expressing EGFP-Fiber-2 fusion protein and its efficient protective efficacy against lethal challenge with FAdV-4. Compared with the inactivated FAdV-4 vaccine, FA4-EGFP could induce neutralizing antibodies with higher titers earlier. However, the molecular basis for the attenuation of FA4-EGFP needs to be further elucidated. Does the fusion protein of EGFP-Fiber-2 expressed in FA4-EGFP affect the interaction between Fiber-2 and Penton or other host proteins? Can other foreign antigens replace EGFP to generate dual vaccines against both FAdV-4 and other pathogens? Additionally, except for inducing an efficient humoral immune response with high neutralizing antibodies, the protective efficacy for cellular immunity against FAdV-4 for FA4-EGFP also needs to be further investigated.

## Supplementary Information

**Additional file 1. Complete sequence of EGFP_fiber-2.**

## Data Availability

The datasets used and/or analyzed during the current study are available from the corresponding author on reasonable request.

## Abbreviations

FAdV-4: Fowl adenovirus serotype 4
HHS: Hydropericardium-hepatitis syndrome
IBH: Inclusion body hepatitis
GEU: Gizzard erosion and ulceration
LMH: Leghorn male hepatoma
mAb: Monoclonal antibody
HA: Homologous arm
hpi: Hour post-infection
dpi: Day post-infection
dpc: Day post-challenge
NC: Nitrocellulose
RT: Room temperature
DMEM: Dulbecco modified Eagle medium
FBS: Fetal bovine serum
IFA: Indirect immunofluorescent assay
