# Supplementary material for: A novel fiber-2-edited live attenuated vaccine candidate against the highly pathogenic serotype 4 fowl adenovirus
Source: Vet Res. 2021 Feb 27;52:35. doi: 10.1186/s13567-021-00907-z (PMC7912893; doi:10.1186/s13567-021-00907-z)
Supplement: Supplementary file 1 — Additional file 1. Complete sequence of EGFP_fiber-2. [file 13567_2021_907_MOESM1_ESM.docx]

Complete sequence of EGFP_fiber-2:

ATGGTGAGCAAGGGCGAGGAGCTGTTCACCGGGGTGGTGCCCATCCTGGTCGAGCTGGACGGCGACGTAAACGGCCACAAGTTCAGCGTGTCCGGCGAGGGCGAGGGCGATGCCACCTACGGCAAGCTGACCCTGAAGTTCATCTGCACCACCGGCAAGCTGCCCGTGCCCTGGCCCACCCTCGTGACCACCCTGACCTACGGCGTGCAGTGCTTCAGCCGCTACCCCGACCACATGAAGCAGCACGACTTCTTCAAGTCCGCCATGCCCGAAGGCTACGTCCAGGAGCGCACCATCTTCTTCAAGGACGACGGCAACTACAAGACCCGCGCCGAGGTGAAGTTCGAGGGCGACACCCTGGTGAACCGCATCGAGCTGAAGGGCATCGACTTCAAGGAGGACGGCAACATCCTGGGGCACAAGCTGGAGTACAACTACAACAGCCACAACGTCTATATCATGGCCGACAAGCAGAAGAACGGCATCAAGGTGAACTTCAAGATCCGCCACAACATCGAGGACGGCAGCGTGCAGCTCGCCGACCACTACCAGCAGAACACCCCCATCGGCGACGGCCCCGTGCTGCTGCCCGACAACCACTACCTGAGCACCCAGTCCGCCCTGAGCAAAGACCCCAACGAGAAGCGCGATCACATGGTCCTGCTGGAGTTCGTGACCGCCGCCGGGATCACTCTCGGCATGGACGAGCTGTACAAGCTCCGGGCCCCTAAAAGAAGACATTCCGAAAACGGGAAGCCCGAGACCGAAGCGGGACCTTCCCCGGCTCCAATCAAGCGCGCCAAACGCATGGTGAGAGCATCCCAGCTTGACCTGGTTTATCCTTTCGATTACGTGGCCGACCCCGTCGGAGGGCTCAACCCGCCTTTTTTGGGAGGCTCAGGACCCCTAGTGGACCAGGGCGGACAGCTTACGCTCAACGTCACCGATCCCATCATCATCAAGAACAGATCGGTGGACTTGGCCCACGACCCCAGTCTCGATGTCAACGCCCAAGGTCAACTGGCGGTGGCCGTTGACCCCGAAGGGGCCCTGGACATCACCCCCGATGGACTGGACGTCAAGGTCGACGGAGTGACCGTAATGGTCAACGATGACTGGGAACTGGCCGTAAAAGTCGACCCGTCCGGCGGATTGGATtCCACCGCGGGTGGACTGGGGGTCAGCGTGGACGACACCTTGCTCGTGGATCAGGGAGAACTGGGCGTACACCTCAACCAACAAGGACCCATCACTGCCGATAGCAGTGGTATCGACCTCGAGATCAATCCTAACATGTTCACGGTCAACACCTCGACCGGAAGCGGAGTGCTGGAACTCAACCTAAAAGCGCAGGGAGGCATCCAAGCCGACAGTTCGGGAGTGGGCGTTTCCGTGGATGAAAGCCTACAGATTGTCAACAACACTCTGGAAGTGAAACCGGATCCCAGCGGACCGCTTACGGTCTCCGCCAATGGCCTAGGGCTGAAGTACGACACTAATACCCTAGCGGTGACCGCGGGCGCTTTAACCGTGGTCGGAGGGGGGAGCGTCTCCACACCCATCGCTACTTTTGTCTCGGGAAGTCCCAGCCTCAACACCTACAATGCCACGACCGTCAATTCCAGCGCGAACGCCTTCTCTTGCGCCTACTACCTTCAACAGTGGAACATACAGGGGCTCCTTGTTACCTCCCTCTACTTGAAATTGGACAGCGCCACCATGGGGAATCGCCCTGGGGACCTCAACTCCGCCAATGCCAAATGGTTCACCTTTTGGGTGTCCGCCTATCTCCAGCAATGCAACCCCTCCGGGATTCAAGCGGGAACGGTCAGCCCCTCCACCGCCACCCTCACGGACTTTGAACCCATGGCCAATAGGAGCGTGACCAGCCCATGGACGTACTCGGCCAATGGATACTATGAACCATCCATCGGGGAATTCCAAGTGTTCAGCCCGGTGGTAACAGGTGCCTGGAACCCGGGAAACATAGGGATCCGCGTCCTCCCCGTGCCGGTTTCGGCCTCCGGAGAGCGATACACCCTTCTATGCTATAGTCTGCAGTGCACGAACGCGAGCATTTTTAATCCAAACAACAGCGGAACCATGATCGTGGGACCCGTGCTCTACAGCTGTCCAGCGGCCTCCCTCCCGTAA
